# Improving Multi-Epitope Long Peptide Vaccine Potency by Using a Strategy that Enhances CD4+ T Help in BALB/c Mice

**DOI:** 10.1371/journal.pone.0142563

**Published:** 2015-11-10

**Authors:** Haniyeh Ghaffari-Nazari, Jalil Tavakkol-Afshari, Mahmoud Reza Jaafari, Sahar Tahaghoghi-Hajghorbani, Elham Masoumi, Seyed Amir Jalali

**Affiliations:** 1 Immunogenetic and Cell Culture Department, Immunology Research Center, School of Medicine, Mashhad University of Medical Sciences, Mashhad, Iran; 2 Biotechnology Research Center, Nanotechnology Research Center, School of Pharmacy, Mashhad University of Medical Sciences, Mashhad, Iran; 3 Department of Immunology, Medical School, Shahid Beheshti University of Medical Sciences, Tehran, Iran; Kermanshah University of Medical Sciences, ISLAMIC REPUBLIC OF IRAN

## Abstract

Peptide-based vaccines are attractive approaches for cancer immunotherapy; but the success of these vaccines in clinical trials have been limited. Our goal is to improve immune responses and anti-tumor effects against a synthetic, multi-epitope, long peptide from rat Her2/neu (rHer2/neu) using the help of CD4+ T cells and appropriate adjuvant in a mouse tumor model. Female BALB/c mice were vaccinated with P_5+435_ multi-epitope long peptide that presents epitopes for cytotoxic T lymphocytes (CTL) in combination with a universal Pan DR epitope (PADRE) or CpG-oligodeoxynucleotides (CpG-ODNs) as a Toll-like receptor agonist adjuvant. The results show that vaccination with the multi-epitope long peptide in combination with the PADRE peptide and CpG-ODN induced expansion of subpopulations of CD4+ and CD8+ cells producing IFN-γ, the average tumor size in the vaccinated mice was less than that of the other groups, and tumor growth was inhibited in 40% of the mice in the vaccinated group. The mean survival time was 82.6 ± 1.25 days in mice vaccinated with P_5+435_ + CpG+ PADRE. Our results demonstrate that inclusion of PADRE and CpG with the peptide vaccine enhanced significant tumor specific-immune responses in vaccinated mice.

## Introduction

Breast cancer is one of the most common malignancies in women and the second leading cause of cancer deaths among women worldwide [1]. Amplification and/or overexpression of the Her2/neu protein has been reported in 25–30% of human breast cancers [2]. Her2/neu is a member of the epidermal growth factor receptor family with tyrosine kinase activity [3] and is known as a tumor-associated antigen (TAA) [4]. Overexpression of Her2/neu is associated with aggressive disease and poor prognosis [5]. Although Her2/neu is a self-antigen, antibody and cytotoxic T lymphocyte (CTL) -specific responses against Her2/neu have been detected in some patients with Her2/neu overexpression in breast and ovarian cancers [6, 7];thus, immunological tolerance to Her2 is not absolute and can be overcome [5]. Therefore, Her2/neu is an attractive target for immunotherapeutic approaches [8]. Monoclonal antibodies have demonstrated considerable effects in HER2-positive breast cancer patients. Despite these successes, most metastatic tumors will progress. Therefore a other immunotherapy strategies are needed [9]. Use of Her2-specific peptide-based vaccines is an effective strategy for generating active immune responses to Her2 [10]. Peptide-based vaccines are easily produced, chemically stable, cost effective, non-toxic, and safe [11, 12]. Because CTLs play an important role in the prevention of tumor growth [13], many minimal CTL epitopes derived from TAAs have been identified [14], and numerous peptide-based vaccine investigations have used minimal sequences of MHC class I binding CD8+ Tcell epitopes [15]. Studies have shown peptide-based vaccine induction of CTL responses and anti-tumor protection [16]. In contrast, less encouraging results have been obtained in cancer patients in the clinic [12, 17]. Therefore, it is necessary to improve the effectiveness and potency of peptide vaccines. Multiple approaches have been used to augment the potency of peptide vaccines [18]. Multiepitope long vaccines as one choice are being developed to improve the efficacy of peptide-based vaccines [10, 19]. Several preclinical and clinical models demonstrate that vaccinations with long peptides result in more robust protective immunity capable of improving specific CTL responses than the minimal CTL epitope peptide-based vaccines [20–24]. Vaccination of HPV16-positive advanced or recurrent carcinoma patients with a mix of thirteen E6 and E7 overlapping 25–30 amino acids in an HPV-derived long peptide revealed that it was safe and able to induce HPV-specific responses in 11 of 13 patients [25].

It is well documented that CD4+ T cells play a central role in orchestrating anti-tumor immunity and in priming and maintenance of CD8+ Tcell effector functions. Immune responses have been enhanced by including CD4+ T cell epitopes in peptide vaccines [26, 27]. The presence of a universal T helper epitope such as the pan DR-biding epitope (PADRE) greatly improved antibody immune responses induced by a malaria recombinant antigen vaccine [28]. PADRE is a universal synthetic 13 amino acid peptide that activates CD4+ T cells [29]. Because PADRE binds with high affinity to 15 of the 16 most common human HLA-DR types, and with moderate-to-high affinity to mouse I-A^b/d^ and I-E^b/d^ MHC haplotypes, it provides effective CD4+ T cell responses [30, 31], and likely, PADRE can overcome the problems caused by polymorphism of HLA-DR molecules in the population [32]. A proliferation assay showed PADRE to be 100-fold more potent than other universal T helper epitopes such as the tetanus toxin-derived universal epitope [33]. In addition, human clinical studies have shown PADRE to be safe and well tolerated [34]. A study in a murine model using an E7 peptide-based vaccine in combination with PADRE and a Toll-like receptor 3 (TLR3) agonist indicated better CTL responses and anti-tumor protection than the vaccine without the T helper epitope [35]. To enhance immune responses by peptide-based vaccines and induce CD4+ T cell help, an appropriate adjuvant will be required. Bacterial DNA often have strong immunostimulatory effects that can be mimicked by synthetic oligodeoxynucleotides with unmethylated CpG motifs (CpG-ODNs) [36]. CpG-ODNs interact with TLR-9 and induce Th1-based immune responses [37, 38]. These can also stimulate monocytes, macrophages, and B cells, and induce activation and maturation of dendritic cells (DCs) [39]. Studies have shown that the addition of CpG as a vaccine adjuvant can enhance immune responses [40–42].

Our aim in this study was to improve CTL immune responses using a multi-epitope long peptide combined with PADRE as a universal T-helper cell epitope and CpG-ODN as an adjuvant.

## Materials and Methods

### Mice

Female six-to-eight-week-old BALB/c mice were purchased from the Pasteur Institute (Tehran, Iran). All mice were maintained under pathogen-free conditions and allowed to acclimate to the animal facility of the BuAli Research Institute (Mashhad, Iran) for one week before beginning the experiments. Experiments were conducted in accordance with the proposal and were approved by the Institute Ethical Committee and Research Advisory Committee of Mashhad University of Medical Sciences.

### Cell line

The TUBO cloned cell line is a Her2/neu-overexpressing murine tumor model derived from a lobular carcinoma that arose spontaneously from BALB/c mice transgenic for the rat Her2/neu (BALB/neuT) oncogene [4]. TUBO cells grow progressively in normal BALB/c mice and give rise to lobular carcinoma, which is histologically similar to that seen in BALB/neuT mice [43]. TUBO cells were kindly provided by Dr. Pier-Luigi Lollini (Department of Clinical and Biological Sciences, University of Turin, Orbassano, Italy). The cells were cultured in Dulbecco's Modified Eagle Medium (DMEM) supplemented with 100U/ml penicillin, 100 μg/ml streptomycin, and 20% heat-inactivated fetal bovine serum (FBS), at 37°C with 5% CO2.

### Peptides

We used the P_5+ 435_ multiepitope peptide consisting of two CTL epitopes, P5 and P435, each with a length of 21 amino acids, which in a previous study were designed by in silico analysis and able to induce rat Her2/neu (rHer2/neu) -specific CTL immune responses. These peptides contain several motifs restricted by MHC class I molecules of BALB/c (H2-Dd, H2-Kd, and H2-Ld) mice [44]. In this study, the P_5+435_ multiepitope long peptide was prepared with a two-arginine residue sequence (RR) as linker between the P_5_ and P_435_ peptides. The R residues were introduced as a protease-sensitive linker, such that once the vaccine was internalized by DCs, intracellular proteases would cleave at the RR bipeptide and separate P_5_ from P_435_, thus enhancing processing and presentation of the epitopes [45, 46]. We also reasoned that many high-affinity CTL epitopes are hydrophobic, making them difficult to dissolve in the aqueous buffers necessary for immunization and biological testing. Hence, polar side chains, such as those of arginine, would increase the solubility of these peptides [47]. The 44 amino acid P_5+435_ synthetic peptide (ELAAWCRWGFLLALLPPGIAG**RR**IRGRILHDGAYSLTLQGLGIH) and PADRE (AKFVAAWTLKAAA) were synthesized and characterized by analytical high-performance liquid chromatography (HPLC) and mass spectrometry by Sbs Bio Inc (Beijing, China) with purity > 95%. Lyophilized peptides were dissolved in dimethylsulfoxide (DMSO) and stored at -20°C until further use. The immunostimulatory synthetic CpG-ODN 1826 (5-TCCATGA**CG**TTCCTGA**CG**TT-3), optimized for stimulation of the mouse immune system and containing two CpG motifs, was used in this work [42, 44]. Synthetic ODNs were prepared with a nuclease-restricted phosphorothioate backbone by Microsynth (Balgach, Switzerland).

### Immunization

Six groups of female BALB/c mice (n = 9/group) were immunized by subcutaneous (s.c.) injections to the flank with 100 μg of the P_5+435_ peptide alone or combined with 25 μg of CpG-ODN 1826 or 25μg of CpG-ODN 1826 plus 50 μg of PADRE in a total volume of 100 μl/mouse, three times at 14 day intervals. Mice similarly vaccinated with phosphate-buffered saline (PBS), PADRE alone, and mixed CpG + PADRE were used as controls. Two weeks after the last immunization, three mice per group were euthanized and their spleens were collected to evaluate cellular immune responses.

### Enzyme-linked immunospot (ELISpot) assay

Two weeks after the last immunization, single-cell splenocyte suspensions were prepared. Interferon-gamma (IFN-γ) production was assessed by a mouse IFN-γ ELISpot assay kit (U-Cy Tech, Utrecht, The Netherlands) according to the manufacturer’s protocol. Flat-bottom, 96-well ELISPOT plates were coated with 50 μl/well of anti-mouse IFN-γ capture antibody, and incubated overnight at 4°C. The plates were washed with PBS/0.05% Tween 20 and blocked with 200 μl/well of blocking buffer for 1 h at 37°C. After removal of the blocking solution, splenocytes were added to wells at concentrations of 10^5^, 3× 10^5^, and 3× 10^4^ cells/well in final volumes of 200μl with RPMI 1640 containing 10 μg/ml of each peptide or a 0.3 μl/ml mixture of 50 ng/ml phorbol 12-myristate13-acetate (PMA) and 500 ng/ml ionomycin (IO) as positive controls, and media alone was used as a negative control. After 24 h at 37°C with 5% CO_2_, plates were washed with PBS/0.05% Tween 20 and then incubated for 24 h at 37°C with 5% CO_2_ with 100 μl/well of biotinylated antibody. After washing, 100 μl of anti-biotin antibody were added to the wells and incubated for 1 h at 37°C with 5% CO_2_. Plates were washed and spots were developed by incubation for 10–30 min with 35 μl/well of substrate at room temperature in the dark. Spots were counted with the Kodak 1D software package (Version 3.5, Eastman Kodak, Rochester, New York). The mean number of spots ± SD in triplicate wells was calculated and expressed as spot-forming units (SFU) per 10^6^ splenocytes.

### Surface and intracellular staining

For flow cytometry, splenocytes were stimulated with 0.7 μl/ml of 50 ng/ml PMA and 500 ng/ml IO, and then 1μg/ml of berefeldinA was added to retain the cytokine in the cytoplasm. After incubation for 4 h at 37°C with 5% CO_2_, these cells were stained for CD4 or CD8 surface markers using anti-CD4-PEcy5 or anti-CD8-PEcy5 antibodies (all from BD Biosciences) in separate tubes and incubated for 30 min at 4°C in the dark,. For IFN-γ and IL-4 intracellular staining after fixation and permeabilization using a Cytofix/Cytoperm^™^ Plus Fixation/Permeabilization Kit with BD Golgi PlugTM (cat no.555028), cells were stained with anti-IFN-γ-FITC and anti-IL-4-PE antibodies and incubated for 30 min at 4°C in the dark. Stained cells were analyzed using FACSCalibur^™^ (BD Biosciences).

### Prophylactic model of TUBO challenge

Fourteen days after the last immunization, six mice per group were challenged by s.c. injections on the shaved right flank with 50 μl of a single-cell suspension of 5× 10^5^ TUBO cells. Mice were observed weekly for 80 days to monitor tumor growth and tumors were measured with calipers. Tumor masses were measured in three orthogonal diameters, and volumes were determined. Mice with no evidence of tumors during the observation period were classed as tumor-free, mice with tumor masses with mean diameters > 3 mm were classed as tumor bearers. The mice were monitored for up to 80 days unless one of the following conditions for euthanization was met: (a) body weight dropped below 15% of their initial mass, (b) the tumor masses were greater than 20 mm in any dimension, (c) the mice became lethargic or sick and unable to feed. To reduce suffering during euthanizations, each mouse was anesthetized by an i.p. injection of 100 μl/10 g mouse body weight of a ketamine/xylazine mixture containing1 ml of 100mg/ml ketamine and 0.5 ml of 20mg/ml xylazine diluted in 8.5 ml of sterile injectable saline. Mice were euthanized by cervical dislocation. All surviving mice were euthanized on day 80. Five mice in the control group were euthanized by day 80 because of tumor progression.

### Statistical Analysis

The significance of the differences between groups was determined with the One-way ANOVA statistical test using GraphPad Prism version 5 (GraphPad Software, San Diego, CA). Tumor sizes data were analyzed by the repeated measures ANOVA test. The tukey test was used to compare the means of different groups. The log-rank test using GraphPad Software and the Fleming-Harrington test using STATA Software were used to analyze survival between groups. P < 0.05 was considered significant.

## Results

### Mice vaccinated with the P_5+435_ long peptide combined with the PADRE peptide and CpG produced the greatest amount of IFN-γ

**Fig 1:**
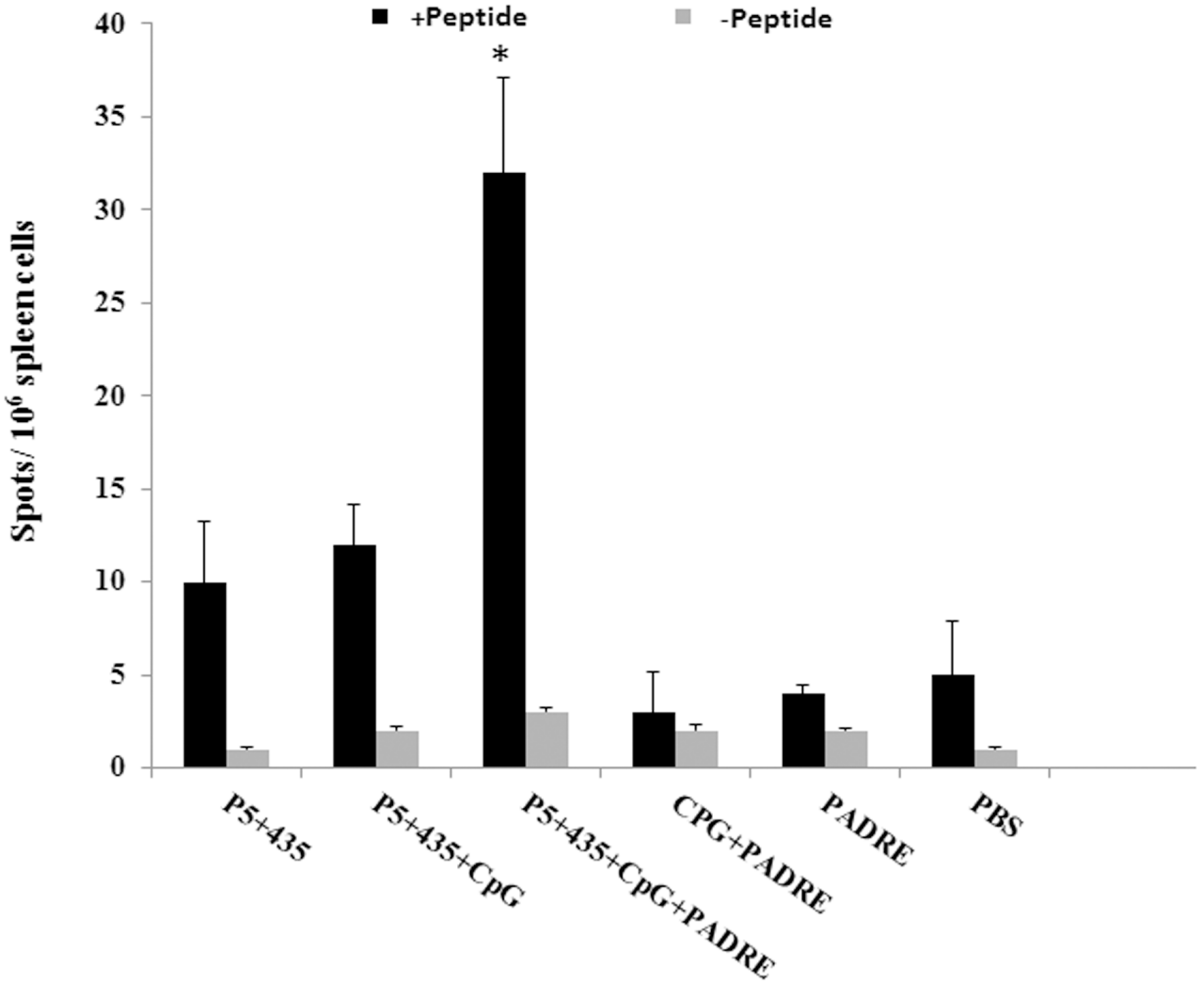
Evaluation of the amount of IFN-γ produced in vaccinated mice. Nine BALB/c female mice per group were vaccinated three times subcutaneously with 100 μg/mouse of P_5+435_ long peptide, P_5+435_ in combination with CpG, or in combination with both 50 μg/mouse of PADRE and 25 μg/mouse of CpG. Two weeks after the last vaccination, splenocytes from three mice from each group were collected and activated with the long peptide. Immune responses were then determined using an IFN-γ ELISpot assay. The data indicate the mean ± SD. (n = 3). * denotes significant difference from all other groups (P < 0.001).

To determine the immune response in mice vaccinated with P_5+435_ in combination with the PADRE peptide and CpG, we performed IFN-γ ELISpot assays. Mice were vaccinated with the P_5+435_ long peptide, the long peptide + CpG, or the long peptide + CpG + PADRE. Control groups were vaccinated with PADRE alone, PADRE + CpG, or PBS. Fourteen days after the last vaccination, three mice from each group were euthanized and splenocyte IFN-γ production was measured by the IFN-γ ELISpot assay. As shown in Fig 1, the mice vaccinated with the P_5+435_ long peptide + PADRE + CpG produced significantly more IFN-γ than the mice vaccinated with P_5+435_ alone, P_5+435_ + CpG, or controls (P <0.001).

### Immunization with the P_5+435_ long peptide in combination with PADRE and CpG increased the percentage of CD8 T cells that produce IFN-γ

**Fig 2:**
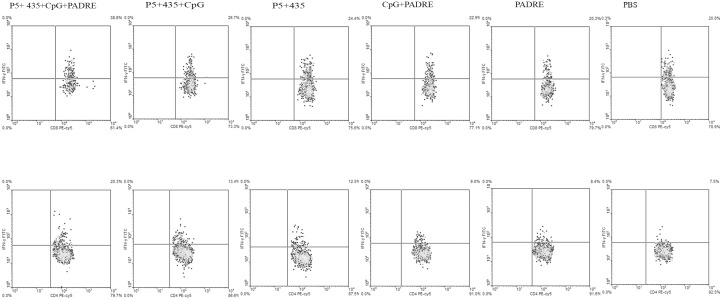
The percentages of IFN-γ producing CD4+ and CD8+ T cells. Flow cytometry data were also analyzed according to the percentage of cytokine-producing cells and dot plots were drawn for each vaccinated group. Quadrants showing dot plot of CD8 and CD4 cells producing IFN-γ percentage. Spleen cells were analyzed using a gating strategy to exclude debris and identify CD4+ and CD8+ T cells. The subsequent analysis was on CD8+ or CD4+ gates to describe IFN-γ-producing T cells.

**Fig 3:**
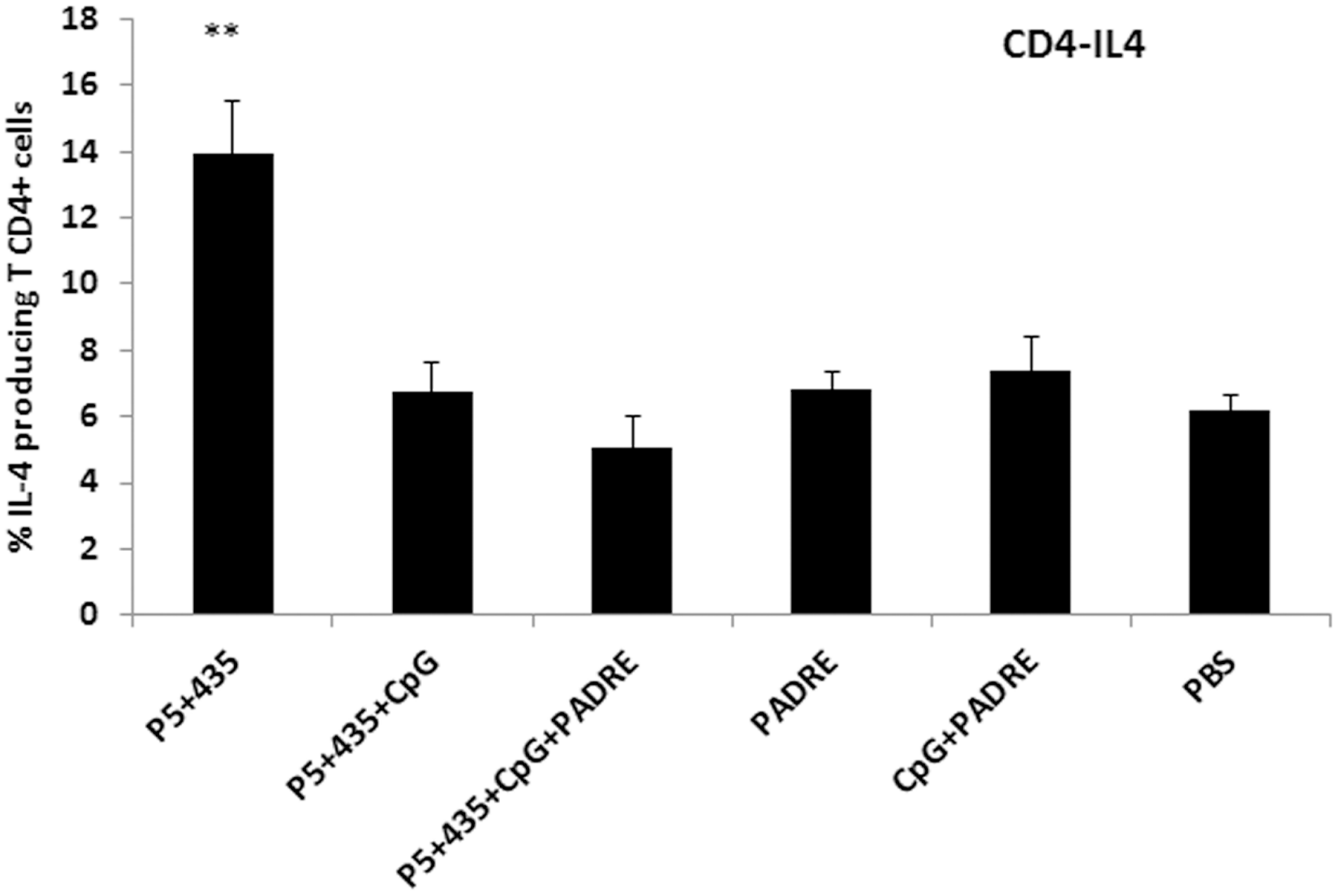
The percentages of IL-4-producing *CD4*+ T cells. Fourteen days after the last vaccination three mice per group were euthanized, and splenocytes were collected and characterized for CD4+ T cells using intracellular IL-4 staining followed by flowcytometry analysis. Data represent mean ± SEM (n = 3). ** denote significant differences from controls and all other groups, respectively.

To evaluate subpopulations of CD4+ and CD8+ T cells induced in vaccinated mice, two weeks after the last immunization, splenocytes from three mice from each group were harvested and characterized by flow cytometry. The cells were stained for CD4, CD8, IFN-γ, and IL-4. The percentage of cytokine-producing cells were obtained for each group. As shown in Fig 2, both CD4+ and CD8+ T cells from mice that received P_5+435_ + PADRE + CpG produced significantly greater amounts of IFN-γ than the control groups. We also found that CD4+ T cells from mice vaccinated with P_5+435_ alone produced significantly more IL-4 than the other groups (P = 0.009) (Fig 3).

### Vaccination with the P_5+435_ long peptide + PADRE peptide + CpG inhibited tumor growth in BALB/c mice challenged with live TUBO tumor cells

**Fig 4:**
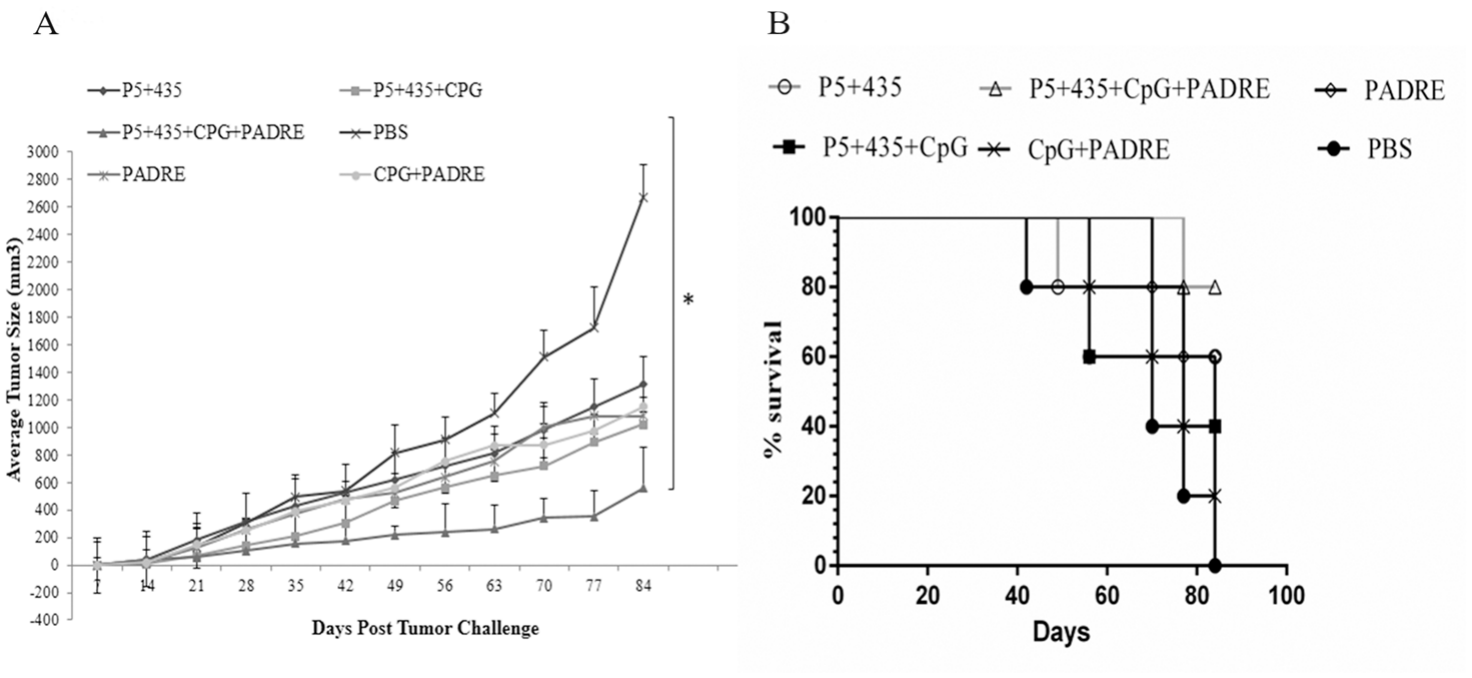
In vivo antitumor effects experiments. Six mice/group were immunized three times with P_5+435_ long peptide alone, long peptide + CpG, or long peptide + PADRE + CpG. Control mice were immunized with PADRE, PADRE + CpG, or PBS. After 14 days the mice were challenged subcutaneously with 5× 10^5^ live TUBO cells. (A) Tumors were measured weekly and sizes were recorded. The values are means of tumor size and error bars indicate SD. (B) The survival times of the mice were analyzed by log-rank (P = 0.117) and Fleming-Harrington tests (P = 0.058) for 80 days. * denotes significant difference from PBS (P < 0.05).

To determine whether the immune responses induced by vaccination were potent enough to generate antitumor protection, we used BALB/c mice in the prophylactic model. Fourteen days after the last vaccination, six mice from each group were challenged subcutaneously with 5×10^5^ live TUBO cells. Fig 4A shows that tumor growth in the mice that received the P_5+435_ long peptide in + PADRE + CpG was slower than that of the other groups (P = 0.019). Although the tumors grew for a few weeks, the tumors growth in 33 percent of the mice vaccinated with P_5+435_ long peptide + PADRE peptide + CpG were completely prevented, and these mice remained tumor-free until the end of the 80-day experimental period. Survival was higher in this group than in the other groups but not statistically significant (P = 0.11) (Fig 4B).

## Discussion

Our study had two goals, first to design a multi-epitope long peptide, and second, to increase immune responses generated by the long peptide with the addition of PADRE and a CpG adjuvant.

Various methods have been used to design multi-epitope long peptides. A typical approach consists of linking minimal epitopes together. Some studies showed that spacers or linkers must be used between epitopes to achieve optimal processing while others did not [48]. Another approach for multi-epitope long peptide design consists of applying “natural” peptides of more than 17 amino acids, because many previous studies reported that peptides with more than 17 amino acids require proteasome processing and presentation of the epitopes to CTLs [49].

Here, we combined both methods and used two 21 amino acid “natural” peptides from the CTL epitope of rHer2/neu protein described in a previous study [44, 50]. The two peptides were linked by an arginine linker. Thus the 44 amino acid peptide can be internalized, processed, and presented by antigen-presenting cells (APCs) and simultaneously stimulate different T cell clones. Given that both peptides are from CTL epitopes of the rHer2/neu protein, we decide to apply a universal T helper epitope to recruit CD4+ T cell help in immune responses induced by the long peptide. PADRE was used to induce CD4+ T cell help and augment the immune responses generated by the long peptide.

In the present study, We compared the effectiveness of different vaccines on immune responses. Effective immune responses were obtained with peptide alone, peptide combined with CpG, and peptide combined with CpG and PADRE. The mice immunized with the P_5+435_ multi-epitope long peptide vaccine, in combination with PADRE and the CpG adjuvant, showed the strongest specific CTL immune responses of all the immunized groups, had the longest survival times, and were the most tumor-resistant against the Her2/neu-expressing TUBO cell line.

We showed that CD4+ and CD8+ T cells from mice immunized with the P_5+435_ multi-epitope long peptide-based vaccine combined with PADRE and CpG produced more IFN-γ than T cells from mice vaccinated with the peptide alone or the peptide vaccine + CpG. CD4+ T cells play a central role in initiation and regulation of different aspects of immune responses. In addition, CD4+ T cells are essential for the priming of tumor-specific CTLs and maintaining anti-tumor responses [26]. We believe the CD4+ T cells were induced due to the presence of the PADRE peptide and likely contributed to the increased expression of IFN-γ. Our results are consistent with other studies that used peptide- and DNA-based vaccines with PADRE [35, 51].

Another important reason for the enhanced immune response and anti-tumor effects in mice vaccinated with the multi-epitope long peptide + PADRE + CpG is likely related to the use of CpG as an adjuvant. As previously mentioned, synthetic ODNs that contain immunostimulatory CpG motifs trigger an immunomodulatory cascade that involves B and T cells, natural killer cells, and professional APCs [52]. CpG-ODNs used as vaccine adjuvants are rapidly internalized by immune cells and interact with TLR9 in endocytic vesicles. CpG-ODNs can improve APC function and promote the induction of antigen-specific adaptive immune responses by supporting the development of Th1- rather than Th2-type immune responses. In the mice were vaccinated with long peptide + CpG tumors were smaller than those in the other groups but these not statistically significant. The CpG positive effects in improving the potency of immune response were shown in different studies when used as a cancer vaccine adjuvant. The CpG effects in our study were not statistically significant but this was more than long peptide alone and PBS groups.

We also found that CD4+ T cells from mice immunized with the long peptide + CpG or CpG + PADRE produced less IL-4 than mice immunized with the long peptide alone. Although tumors in mice immunized with the long peptide alone were smaller than those in mice immunized with PBS, but long peptide antitumor effects were weak in comparison with the mice that received the long peptide + CpG + PADRE. Almost 33 percent of the mice immunized with the long peptide + PADRE + CpG had no tumor growth throughout the experiment. Our results showed that use of the long peptide alone increases subpopulations of IFN-γ-producing CD4+ and CD8+ T cells, as well as a subpopulation of IL-4-producing CD4+ cells. Given that we identified CD4+ and CD8+ T cells producing IFN-γ and CD4+ T cells producing IL-4, we detected both Th1 and Th2 profiles in the spleens. In fact, when we used the long peptide without CpG and PADRE both Th1 and Th2 immune responses were induced. A likely reason appropriate antitumor responses were not achieved in the mice vaccinated with the long peptide alone is because in these mice the immune response was directed toward Th2. The induced Th2 response may be due to decreased potency of an induced Th1 response with weak antitumor activity generated by the long peptide alone.

In summary, our results demonstrate that the long peptide alone stimulated the immune system, but was not completely effective. Addition of the PADRE peptide and CpG adjuvant enhanced the immune response and antitumor effects of the rHer2 multi-epitope long peptide vaccine in immunized mice. PADRE and other strategies that induce CD4+ T cells can be used to improve the efficacy and potency of peptide vaccines.
